# Validation and updating of risk models based on multinomial logistic regression

**DOI:** 10.1186/s41512-016-0002-x

**Published:** 2017-02-08

**Authors:** Ben Van Calster, Kirsten Van Hoorde, Yvonne Vergouwe, Shabnam Bobdiwala, George Condous, Emma Kirk, Tom Bourne, Ewout W. Steyerberg

**Affiliations:** 1 KU Leuven Department of Development and Regeneration, Herestraat 49 box 805, 3000 Leuven, Belgium; 2 Department of Public Health, Erasmus MC, Wytemaweg 80, 3015 CN Rotterdam, The Netherlands; 3 Open Analytics NV, Jupiterstraat 20, 2600 Antwerp, Belgium; 4 Queen Charlotte’s and Chelsea Hospital, Imperial College, Du Cane Road, London, W12 0HS UK; 5 Acute Gynaecology, Early Pregnancy and Advanced Endosurgery Unit, Nepean Medical School, Nepean Hospital, University of Sydney, Kingswood, NSW Australia; 6 North Middlesex University Hospital, Sterling Way, London, N18 1QX UK; 7 Department of Obstetrics and Gynecology, University Hospitals Leuven, Herestraat 49 box 7003, Leuven, Belgium

**Keywords:** Calibration, Discrimination, Model updating, Multicategory outcome, Multinomial logistic regression, Prediction models, Risk models

## Abstract

**Background:**

Risk models often perform poorly at external validation in terms of discrimination or calibration. Updating methods are needed to improve performance of multinomial logistic regression models for risk prediction.

**Methods:**

We consider simple and more refined updating approaches to extend previously proposed methods for dichotomous outcomes. These include model recalibration (adjustment of intercept and/or slope), revision (re-estimation of individual model coefficients), and extension (revision with additional markers). We suggest a closed testing procedure to assist in deciding on the updating complexity. These methods are demonstrated on a case study of women with pregnancies of unknown location (PUL). A previously developed risk model predicts the probability that a PUL is a failed, intra-uterine, or ectopic pregnancy. We validated and updated this model on more recent patients from the development setting (temporal updating; *n* = 1422) and on patients from a different hospital (geographical updating; *n =* 873). Internal validation of updated models was performed through bootstrap resampling.

**Results:**

Contrary to dichotomous models, we noted that recalibration can also affect discrimination for multinomial risk models. If the number of outcome categories is higher than the number of variables, logistic recalibration is obsolete because straightforward model refitting does not require the estimation of more parameters. Although recalibration strongly improved performance in the case study, the closed testing procedure selected model revision. Further, revision of functional form of continuous predictors had a positive effect on discrimination, whereas penalized estimation of changes in model coefficients was beneficial for calibration.

**Conclusions:**

Methods for updating of multinomial risk models are now available to improve predictions in new settings. A closed testing procedure is helpful to decide whether revision is preferred over recalibration. Because multicategory outcomes increase the number of parameters to be estimated, we recommend full model revision only when the sample size for each outcome category is large.

## Background

Prior to implementing risk prediction models in clinical practice to assist in patient management, their performance needs to be rigorously validated. Core elements of performance include discrimination (i.e., how well the model discriminates between the different categories) and calibration (i.e., the reliability of the predicted risks) [1, 2]. It is of particular importance to externally validate the model using data collected later in time (temporal validation) and/or in different locations or hospitals (geographical validation) [3, 4]. Disappointing validation results do not necessarily imply that the previously developed prediction model should be discarded, because the model contains crucial information such as which predictors are considered relevant. An attractive alternative is to perform some form of model updating, where we combine information from the previously developed model with new data [5]. This approach has clear practical relevance, because it is often not realistic to expect that a single model will work in all settings, due to differences in patient management protocols and referral patterns across centers and regions, and improvements in care over time. Updating is particularly useful for model validation in settings with different patient populations (e.g., primary vs secondary care), sometimes labeled “domain validation,” because of likely differences in case-mix, event rates, predictor definitions, and measurement methods [6].

Methods to update risk models for dichotomous outcomes focus on recalibration, revision, and extension [1, 5, 7]. Recalibration merely adjusts the model intercept and/or overall slope, where an overall slope adjustment implies a fixed proportional adjustment of all predictor coefficients. Model revision adjusts the individual model coefficients, and model extension refits the model while including additional markers.

Van Hoorde and colleagues assessed how dichotomous recalibration and revision techniques could be extended to multicategory outcomes for which risk estimation was based on a sequence of dichotomous logistic regression models (sequential dichotomous modeling) [8]. The aim of the current paper is to introduce methods to directly update risk models for multicategory outcomes based on multinomial logistic regression, which is the most commonly used method for nominal outcomes. We present recalibration, revision, and extension methods and a statistical test to direct the preferred strategy. We illustrate these methods on a case study.

## Methods

### Case study

This case study aims to guide further follow-up for women with a pregnancy of unknown location (PUL). A PUL involves a pregnant woman whose pregnancy cannot be visualized using transvaginal ultrasound [9, 10]. It is important to estimate the likelihood that the PUL is a failed PUL (FPUL), an ectopic pregnancy (EP), or an intra-uterine pregnancy (IUP) [10]. Potential follow-up strategies are to perform (a) a urinary pregnancy test after 2 weeks if an FPUL is predicted, (b) a repeat ultrasound scan after 1 week if an IUP is predicted, and (c) a repeat ultrasound scan and human chorionic gonadotropin (hCG) assessment after 2 days if an EP is predicted [11]. M4 is a multinomial logistic regression model developed for this purpose that is based on the serum hCG levels at presentation (hCG0) and 48 h later (hCG48) [12]. M4 was developed on data from women recruited at St. George’s Hospital (SGH) in London between March and November 2002 [12]. Among the 197 patients, 109 (55%) had a FPUL, 76 (39%) an IUP, and 12 (6%) an EP (Table 1). M4 has the following predictors: the logarithm of the average of hCG0 and hCG48 (hCGm) and the ratio of hCG48 and hCG0 (hCGr). A centered version of hCGr was used by subtracting the median ratio of 1.17 from all values (hCGrc), and a quadratic effect of hCGrc was included. The linear predictors for FPUL vs IUP (LP_FvsI_) and for EP vs IUP (LP_EvsI_) are

**Table 1:** Descriptive statistics for the case study of multicategory outcome prediction: original development data of model M4 (*n =* 197), the temporal updating data at SGH (*n =* 1422), and the geographical updating data at QCCH (*n =* 873)

	Original development data (SGH) *n =* 197	Temporal updating (SGH) *n =* 1422	Geographical updating (QCCH) *n =* 873
Age (years)	30 (25–33)	31 (26–35)	32 (27–32)
Initial hCG (IU/L)	265 (76–618)	410 (154–941)	530 (197–1563)
hCG ratio	0.80 (0.33–1.99)	1.04 (0.39–2.10)	0.65 (0.34–1.49)
Initial progesterone (nmol/L)	17 (4–66)	21 (4–61)	9 (3–34)
Outcome, *n* (%)			
Failed	109 (55%)	717 (50%)	502 (58%)
IUP	76 (39%)	577 (41%)	245 (28%)
Ectopic	12 (6%)	128 (9%)	126 (14%)

Data are expressed as median (interquartile range) or as N (%). In the temporal updating data, progesterone was missing in 47 patients (3.3%), and in the geographical updating data, progesterone was missing in 109 patients (12.5%)

*SGH* St George’s Hospital, *QCCH* Queen Charlotte and Chelsea’s Hospital, *IUP* intra-uterine pregnancy, *hCG* human chorionic gonadotropin

1 $$ \left\{\begin{array}{c}\hfill {\mathrm{LP}}_{\mathrm{FvsI}}= log\left(\frac{P_{\mathrm{FPUL}}}{P_{\mathrm{IUP}}}\right)=5.88-1.18\times log\left(\mathrm{hCGm}\right)-5.56\times \mathrm{hCGrc}+2.05\times {\mathrm{hCGrc}}^2\hfill \\ {}\hfill {\mathrm{LP}}_{\mathrm{EvsI}}= log\left(\frac{P_{\mathrm{EP}}}{P_{\mathrm{IUP}}}\right)=0.39-0.06\times log\left(\mathrm{hCGm}\right)-0.26\times \mathrm{hCGrc}-3.93\times {\mathrm{hCGrc}}^2\hfill \end{array}\right. $$

The predicted risks for each outcome category are obtained as

2 $$ \left\{\begin{array}{c}\hfill {P}_{\mathrm{FPUL}}=\frac{exp\left({\mathrm{LP}}_{\mathrm{FvsI}}\right)}{1 + exp\left({\mathrm{LP}}_{\mathrm{FvsI}}\right) + exp\left({\mathrm{LP}}_{\mathrm{EvsI}}\right)}\hfill \\ {}\hfill \hfill \\ {}\hfill {P}_{\mathrm{IUP}}=\frac{1}{1 + exp\left({\mathrm{LP}}_{\mathrm{FvsI}}\right) + exp\left({\mathrm{LP}}_{\mathrm{EvsI}}\right)}\hfill \\ {}\hfill \hfill \\ {}\hfill {P}_{\mathrm{EP}}=\frac{exp\left({\mathrm{LP}}_{\mathrm{EvsI}}\right)}{1 + exp\left({\mathrm{LP}}_{\mathrm{FvsI}}\right) + exp\left({\mathrm{LP}}_{\mathrm{EvsI}}\right)}\hfill \end{array}\right. $$

Here, we validate and update the M4 model using more recent data from the same setting (temporal updating) and using data from another center (geographical updating). For temporal updating, data from consecutive patients recruited between 2002 and 2007 were used. For geographical updating, data from consecutive patients recruited between 2009 and 2013 at Queen Charlotte and Chelsea’s Hospital (QCCH) in London were used. R version 3.2.0 (www.r-project.org) was used for the statistical analysis.

After applying the exclusion criteria (see the Appendix), data from 1422 (88%) patients were available at SGH and data from 873 (80%) women at QCCH. At SGH, there were 717 (50%) FPUL, 577 (41%) IUP, and 128 (9%) EP. The QCCH data contained 502 (58%) FPUL, 245 (28%) IUP, and 126 (14%) EP.

### Updating methods

We implemented seven updating methods (Table 2): two recalibration, three revision, and two extension methods.

**Table 2:** Updating methods for multinomial logistic regression models with the numbers of parameters that are estimated for updating in general and in the case study

Category	Method and description	Number of parameters
		(General = case study)
Original	0—no adjustments	0 = 0
Recalibration	1—intercept recalibration: adjust intercepts	(*k* − 1) = 2
	2—logistic recalibration: adjust intercepts and slopes	*k* × (*k* − 1) = 6
	3—refitting: re-estimation of individual coefficients	(*q* + 1) × (*k* − 1) = 8
Revision	4—penalized refitting using recalibrated coefficients from method 2 as offset	(*k* + *q* + 1) × (*k* − 1) = 14
	5—refitting including functional form: method 3, but hCGr modeled with rcs	(*q*′ + 1) × (*k* − 1) = 8
Extension	6—extension: similar to method 3 but log(progesterone) added	(*q* + *m* + 1) × (*k* − 1) = 10
	7—penalized extension: similar to method 5 but log(progesterone) added	(*k* + *q* + *m* + 1) × (*k* − 1) = 16

*hCGr* human chorionic gonadotropin ratio, *rcs* restricted cubic spline, *k* number of outcome categories, *q* number of variables (including additional nonlinear and interaction terms, but excluding intercepts) in original model, *q* ′ number of variables when changing functional form of one or more predictors, *m* number of variables related to added markers

We denote the number of outcome categories with *k*. In our case study, *k* = 3. Next, we make the distinction between LP_FvsI_ and LP_EvsI_ on the one hand and LP_*x*,FvsI_ and LP_*x*,EvsI_ on the other. LP_FvsI_ and LP_EvsI_ are the linear predictors of the original M4 (Eq. 1), whereas LP_*x*,FvsI_ and LP_*x*,EvsI_ are the updated linear predictors for updating method *x*, where *x* = 1, …, 7. Finally, *q* denotes the number of variables in the model (i.e., including nonlinearity and interaction terms).

#### Reference method

Method 0 applies the original prediction model without any adjustments.

#### Intercept recalibration

The simplest recalibration method (method 1) updates the intercepts of the two linear predictors LP_FvsI_ and LP_EvsI_ of M4:

3 $$ \left\{\begin{array}{c}\hfill {\mathrm{LP}}_{1,\mathrm{FvsI}}={\alpha}_1+{\mathrm{LP}}_{\mathrm{FvsI}}={\alpha}_1+1\times {\mathrm{LP}}_{\mathrm{FvsI}}+0\times {\mathrm{LP}}_{\mathrm{EvsI}}\hfill \\ {}\hfill {\mathrm{LP}}_{1,\mathrm{EvsI}}={\alpha}_2+{\mathrm{LP}}_{\mathrm{EvsI}}={\alpha}_2+0\times {\mathrm{LP}}_{\mathrm{FvsI}}+1\times {\mathrm{LP}}_{\mathrm{EvsI}}\hfill \end{array}\right. $$

Note that multinomial logistic regression models have *k* − 1 linear predictors, with *k* being the number of outcome categories. Per equation in Eq. 3, only one linear predictor corresponds to the outcomes that are compared (the “corresponding” linear predictor), with the other linear predictors labeled as “non-corresponding.” For intercept recalibration, we assume that the coefficients for the corresponding linear predictors are equal to 1 whereas we assume that the non-corresponding linear predictors have a coefficient of 0. This update aims to improve calibration-in-the-large by aligning observed event rates and mean predicted risks [13].

#### Logistic recalibration

For method 2, a multinomial logistic recalibration framework for LP_FvsI_ and LP_EvsI_ is applied [13]:

4 $$ \left\{\begin{array}{c}\hfill {\mathrm{LP}}_{2,\mathrm{FvsI}}={\alpha}_1+{\beta}_1\times {\mathrm{LP}}_{\mathrm{FvsI}}+{\gamma}_1\times {\mathrm{LP}}_{\mathrm{EvsI}}\hfill \\ {}\hfill {\mathrm{LP}}_{2,\mathrm{EvsI}}={\alpha}_2+{\gamma}_2\times {\mathrm{LP}}_{\mathrm{FvsI}}+{\beta}_2\times {\mathrm{LP}}_{\mathrm{EvsI}}\hfill \end{array}\right. $$

Intercepts (*α*), coefficients for corresponding linear predictors (*β*), and coefficients for non-corresponding linear predictors (*γ*) are estimated in order to update the regression coefficients [13]. This method corrects miscalibration of the predicted probabilities from M4, such that there is no general over- or underestimation of risks and such that predicted risks are on average not overly extreme or overly modest. It may be surprising that coefficients for non-corresponding linear predictors are not fixed at zero. Only if the original model is correct for the updating population, all *β*s are 1 and all *γ*s are 0 in Eq. 4. When performing logistic recalibration by setting all *γ*s to 0, there is no unique result: the updated model will be different depending on the choice of reference category in the logistic recalibration model [13]. In the Appendix, we work out the logistic recalibration formula for the case study.

#### Model refitting by re-estimating individual coefficients

Method 3 re-estimates the intercepts and the coefficients of each predictor using the updating data. A straightforward refit using multinomial logistic regression is used:

5 $$ \left\{\begin{array}{c}\hfill {\mathrm{LP}}_{3,\mathrm{FvsI}}={\alpha}_1+{\beta}_{1,1}\times log\left(\mathrm{hCGm}\right)+{\beta}_{1,2}\times \mathrm{hCGrc}+{\beta}_{1,3}\times {\mathrm{hCGrc}}^2\hfill \\ {}\hfill {\mathrm{LP}}_{3,\mathrm{EvsI}}={\alpha}_2+{\beta}_{2,1}\times log\left(\mathrm{hCGm}\right)+{\beta}_{2,2}\times \mathrm{hCGrc}+{\beta}_{2,3}\times {\mathrm{hCGrc}}^2\hfill \end{array}\right. $$

#### Model refitting by penalized estimation of differences with recalibrated coefficients

Method 4 uses the recalibrated linear predictors from method 2 (i.e., LP_2,FvsI_ and LP_2,EvsI_ from Eq. 4) as an offset:

6 $$ \left\{\begin{array}{c}\hfill {\mathrm{LP}}_{4,\mathrm{FvsI}}={\alpha}_1+{\mathrm{LP}}_{2,\mathrm{FvsI}}+{\beta}_{1,1}\times log\left(\mathrm{hCGm}\right)+{\beta}_{1,2}\times \mathrm{hCGrc}+{\beta}_{1,3}\times {\mathrm{hCGrc}}^2\hfill \\ {}\hfill {\mathrm{LP}}_{4,\mathrm{EvsI}}={\alpha}_2+{\mathrm{LP}}_{2,\mathrm{EvsI}}+{\beta}_{2,1}\times log\left(\mathrm{hCGm}\right)+{\beta}_{2,2}\times \mathrm{hCGrc}+{\beta}_{2,3}\times {\mathrm{hCGrc}}^2\hfill \end{array}\right. $$

Adding the linear predictors as offset implies that the changes in the intercepts and predictor coefficients with respect to method 2 are modeled. We used ridge penalization on these changes to shrink coefficients to their recalibrated values in order to prevent an overly complex model leading to too extreme risk predictions [14]. Without such penalization, methods 3 and 4 would be identical.

#### Model refitting including reassessment of functional form

Method 5 re-estimates model coefficients as in method 3, but now the functional form of hCG ratio is updated as well. This is done with a restricted cubic spline (rcs) fit with three knots for the log of hCG ratio [2]:

7 $$ \left\{\begin{array}{c}\hfill {\mathrm{LP}}_{5,\mathrm{FvsI}}={\alpha}_1+{\beta}_{1,1}\times log\left(\mathrm{hCGm}\right)+{\left[{\beta}_{1,2}\ {\beta}_{1,3}\right]}^T\times \mathrm{rcs}\left( log\left(\mathrm{hCGr}\right),\ \mathrm{knots}=3\right)\hfill \\ {}\hfill {\mathrm{LP}}_{5,\mathrm{EvsI}}={\alpha}_2+{\beta}_{2,1}\times log\left(\mathrm{hCGm}\right)+{\left[{\beta}_{2,2}\ {\beta}_{2,3}\ \right]}^T\times \mathrm{rcs}\left( log\left(\mathrm{hCGr}\right),\ \mathrm{knots}=3\right)\hfill \end{array}\right. $$

Because M4 was originally developed on a small dataset, the quadratic effect for hCG ratio may be inadequate or the result of overfitting. Given that hCG ratio is the most important predictor, it is worthwhile to re-assess its functional form. When using rcs with three knots, the number of parameters used to model hCG ratio remains at two. We decide to keep the log-transformation for the average hCG level to limit the overall complexity of the model.

#### Model extension by refitting and adding a novel marker

Method 6 is similar to method 3 but adds the log of the progesterone level at presentation as a novel marker:

8 $$ \left\{\begin{array}{c}\hfill {\mathrm{LP}}_{6,\mathrm{FvsI}}={\alpha}_1+{\beta}_{1,1}\times log\left(\mathrm{hCGm}\right)+{\beta}_{1,2}\times \mathrm{hCGrc}+{\beta}_{1,3}\times {\mathrm{hCGrc}}^2+{\beta}_{1,4}\times log\left(\mathrm{prog}\right)\hfill \\ {}\hfill {\mathrm{LP}}_{6,\mathrm{EvsI}}={\alpha}_2+{\beta}_{2,1}\times log\left(\mathrm{hCGm}\right)+{\beta}_{2,2}\times \mathrm{hCGrc}+{\beta}_{2,3}\times {\mathrm{hCGrc}}^2+{\beta}_{2,4}\times log\left(\mathrm{prog}\right)\hfill \end{array}\right. $$

#### Model extension using penalization

Method 7 is similar to method 4 because the linear predictors from method 2 are used as offset and ridge penalization is used. Penalization then affects (1) the differences in the intercepts and coefficients of log(hCGm), hCGrc, and hCGrc^2^ relative to logistic recalibration and (2) the coefficients for the new predictor:

9 $$ \left\{\begin{array}{c}\hfill {\mathrm{LP}}_{7,\mathrm{FvsI}}={\alpha}_1+{\mathrm{LP}}_{2,\mathrm{FvsI}}+{\beta}_{1,1}\times log\left(\mathrm{hCGm}\right)+{\beta}_{1,2}\times \mathrm{hCGrc}+{\beta}_{1,3}\times {\mathrm{hCGrc}}^2+{\beta}_{1,4}\times log\left(\mathrm{prog}\right)\hfill \\ {}\hfill {\mathrm{LP}}_{7,\mathrm{EvsI}}={\alpha}_2+{\mathrm{LP}}_{2,\mathrm{EvsI}}+{\beta}_{2,1}\times log\left(\mathrm{hCGm}\right)+{\beta}_{2,2}\times \mathrm{hCGrc}+{\beta}_{2,3}\times {\mathrm{hCGrc}}^2+{\beta}_{2,4}\times log\left(\mathrm{prog}\right)\hfill \end{array}\right. $$

### Closed testing procedure

We extend a recently suggested closed testing procedure for updating dichotomous logistic models [15]. A closed testing procedure involves a hierarchical correction for multiple testing to control the overall type I error at the desired alpha level [16, 17]. The procedure for updating dichotomous prediction models is based on the closed testing procedure that was introduced for variable selection with multivariable fractional polynomials [18]. The procedure for multinomial updating compares the original model (method 0) with intercept recalibration (method 1), logistic recalibration (method 2), and straightforward refitting (method 3) (Table 3). To compare four increasingly complex and nested updating methods, the proposed closed testing procedure sequentially assesses the required scope of model updating: from no updating to refitting. The overall null hypothesis to be tested at a predetermined alpha level is that the original model has the same fit as an updated alternative or else that log-likelihood(method 0) = log-likelihood(method 1) = log-likelihood(method 2) = log-likelihood(method 3). The procedure consists of the following steps:

**Table 3:** Description of the closed testing procedure for updating of multinomial logistic regression models

Step	Procedure
1. Original model vs refitting	H0: both models have the same fit, log *L*_original_ = log *L*_refitted_. Test: likelihood ratio test with (*q* + 1) × (*k* − 1)df. Result: if H0 not rejected, choose the original model, else go to step 2.
2. Intercept recalibration vs refitting	H0: both models have the same fit, log *L*_int recal_ = log *L*_refitted_. Test: likelihood ratio test with *q* × (*k* − 1)df. Result: if H0 not rejected, choose intercept recalibration, else go to step 3.
3. Logistic recalibration vs refitting	H0: both models have the same fit, log *L*_logrecal_ = log *L*_refitted_. Test: likelihood ratio test with (*q* − *k* + 1) × (*k* − 1)df. Result: if H0 not rejected, choose logistic recalibration, else choose refitting.

Each test is performed at the prespecified overall alpha level

*H0* null hypothesis, *L* likelihood, *df* degrees of freedom

A. Predetermine the alpha level *α*.
B. Use a likelihood ratio test at *α* to compare the refitted model with the original model. The degrees of freedom is (*q* + 1) × (*k* − 1). If this test is not rejected (*p* value >*α*), there is no statistically significant improvement in fit of the refitted model vs the original model. The procedure stops and the original model is selected. If the test is significant, proceed to the next step.
C. Use a likelihood ratio test at *α* to compare refitting with intercept recalibration. The degrees of freedom is *q* × (*k* − 1). If the test is not rejected, the procedure stops and intercept recalibration is selected. If the test is significant, proceed to the next step.
D. If *q* < *k* (fewer variables than outcome categories), the procedure stops. Else, use a likelihood ratio test at *α* to compare refitting with logistic recalibration. The degrees of freedom is (*q* − *k* + 1) × (*k* − 1). If the test is not rejected, logistic recalibration is selected. If the test is significant, refitting is selected.

### Ridge penalization

Methods 4 and 7 use ridge penalization which fits models using penalized maximum likelihood in order to obtain more stable and shrunken coefficients [14]. In methods 4 and 7, the coefficients of the predictors are shrunken towards the coefficients following logistic recalibration (method 2). Ridge penalization is implemented with the glmnet package in R [19]. The regularization parameter *λ* of the ridge penalty was estimated using 10-fold cross-validation with the deviance as performance criterion.

### Missingness

Extension methods (methods 6 and 7) add the progesterone level at presentation to the model. At SGH 47 (3.3%) women and at QCCH 109 (12.5%) women had a missing value for progesterone. We used single imputation to deal with the missing values for the current illustrative study, although multiple imputation might be preferred to fully account for uncertainty in the imputation process [20]. The log-transformed progesterone level was imputed via fully conditional specification that included age, the logarithm of hCG0, the logarithm of hCG48, and outcome [21].

### Performance evaluation: discrimination and calibration

Performance was evaluated using measures for discrimination and calibration. Optimism-corrected performance was based on bootstrap internal validation (500 bootstrap resamples), as recommended in [22].

The overall discrimination is evaluated using the polytomous discrimination index (PDI), a nominal version of the *c*-statistic [23]. In a set of patients, one from each outcome category (i.e., a set of size *k*), PDI estimates the probability that a patient from a random outcome category is correctly identified by the model. The patient from outcome category *i* is correctly identified in a set if this patient has the highest predicted risk of category *i*. A PDI of 0.7 means that it is estimated that on average 70% of patients from a set is correctly identified. Random performance corresponds to a PDI of 1/*k* [23]. In addition, *c*-statistics for all pairs of categories are calculated using the conditional risk method [24].

The common definition of calibration is that predicted risks should correspond to observed proportions per level of predicted risk: for patients with an estimated risk of event of 0.3, we expect 30% to have/develop this event. To assess calibration, we calculated calibration intercepts and calibration slopes (with 95% CI) [13]. Ideally, we expect calibration intercepts of 0 and a calibration slope of 1. The calibration intercepts indicate whether the risks are systematically overestimated (if <0) or underestimated (if >0). The calibration slopes indicate the presence of too extreme (if <1) or too modest (if >1) risk predictions. For the original model, we derive flexible calibration curves based on vector splines using the VGAM package in R [25]. This is similar to dichotomous calibration plots where observed proportions are based on loess or spline-based analyses [26, 27].

As an overall measure of performance that combines discrimination and calibration, the Brier score was computed. Brier scores were also optimism-corrected.

## Results

### Validation of the original M4 model

**Fig. 1:**
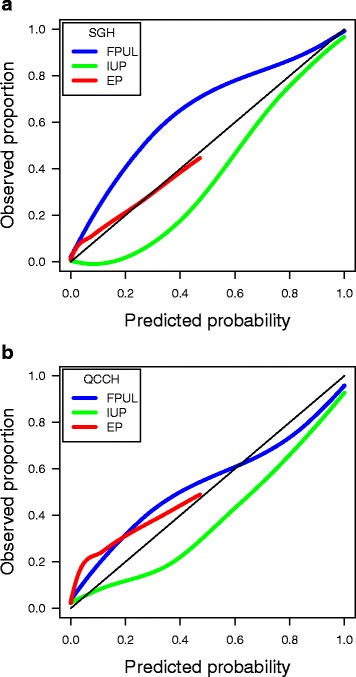
Calibration curves for the original M4 model on the temporal (**a**) and geographical (**b**) updating data

**Fig. 2:**
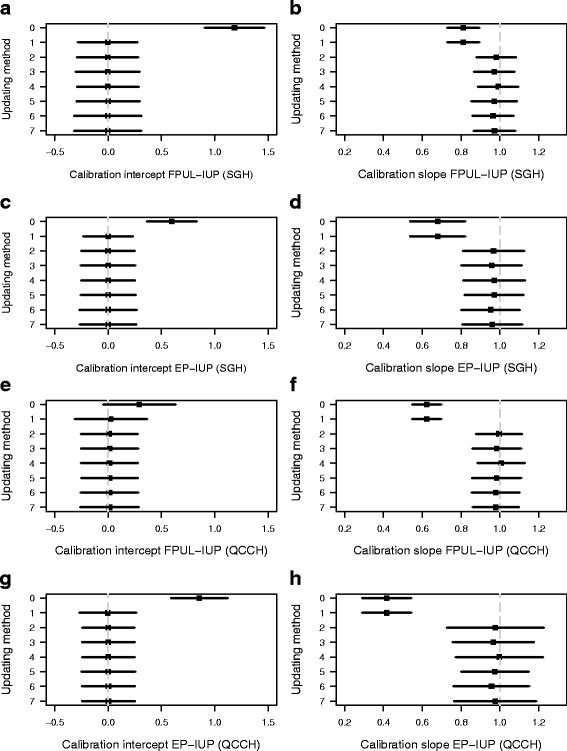
Calibration intercepts and slopes (with 95% CI) after correction for optimism using bootstrapping. Results for temporal updating are shown in **a**–**d** and for geographical updating in **e**–**h**

The original model had very good discrimination. The PDI was 0.87 at temporal updating and 0.80 at geographical updating (Table 4). *c*-statistics were 0.99 and 0.95 for FPUL vs IUP, 0.92 and 0.91 for FPUL vs EP, and 0.89 and 0.84 for IUP vs EP (Table 4). Calibration of predicted risks was poor in both updating settings, as demonstrated by the calibration curves (Fig. 1). The calibration intercepts were 1.19 (FPUL vs IUP) and 0.60 (EP vs IUP) at temporal updating and 0.27 and 0.86 at geographical updating (Fig. 2). The calibration slopes were 0.82 (FPUL vs IUP) and 0.71 (EP vs IUP) at temporal updating and 0.63 and 0.44 at geographical updating (Fig. 2).

**Table 4:** Polytomous discrimination index, pairwise *c*-statistics, and Brier score on the updating data after correction for optimism using bootstrapping

Updating method	PDI	*c*-statistic FPUL-IUP	*c*-statistic FPUL-EP	*c*-statistic IUP-EP	Brier
Temporal updating (SGH)					
No updating	0.87 (0.85–0.90)	0.99 (0.98–0.99)	0.92 (0.89–0.94)	0.89 (0.85–0.93)	0.172 (0.155–0.190)
Intercept recalibration	0.87 (0.84–0.89)	0.99 (0.98–0.99)	0.92 (0.89–0.94)	0.89 (0.85–0.92)	0.165 (0.148–0.183)
Logistic recalibration	0.88 (0.85–0.90)	0.99 (0.98–>0.99)	0.93 (0.91–0.95)	0.91 (0.88–0.94)	0.158 (0.143–0.173)
Refitting	0.88 (0.85–0.90)	0.99 (0.99–>0.99)	0.93 (0.91–0.95)	0.91 (0.88–0.94)	0.157 (0.141–0.172)
Penalized refitting	0.88 (0.85–0.90)	0.99 (0.98–>0.99)	0.93 (0.91–0.95)	0.91 (0.88–0.94)	0.158 (0.142–0.172)
Refitting + rcs	0.88 (0.86–0.91)	0.99 (0.98–>0.99)	0.93 (0.91–0.95)	0.92 (0.89–0.95)	0.153 (0.137–0.168)
Extension	0.89 (0.87–0.92)	0.99 (0.99–>0.99)	0.93 (0.92–0.95)	0.93 (0.90–0.95)	0.150 (0.135–0.165)
Penalized extension	0.89 (0.87–0.92)	0.99 (0.99–>0.99)	0.93 (0.92–0.95)	0.93 (0.90–0.95)	0.150 (0.135–0.165)
Geographical updating (QCCH)					
No updating	0.80 (0.77–0.83)	0.95 (0.93–0.97)	0.91 (0.88–0.94)	0.84 (0.79–0.88)	0.286 (0.258–0.314)
Intercept recalibration	0.80 (0.77–0.83)	0.95 (0.93–0.97)	0.91 (0.88–0.94)	0.84 (0.79–0.88)	0.278 (0.247–0.310)
Logistic recalibration	0.80 (0.77–0.83)	0.96 (0.94–0.97)	0.93 (0.90–0.95)	0.84 (0.79–0.88)	0.267 (0.243–0.291)
Refitting	0.80 (0.77–0.83)	0.96 (0.94–0.97)	0.94 (0.92–0.96)	0.84 (0.80–0.88)	0.266 (0.243–0.291)
Penalized refitting	0.80 (0.77–0.83)	0.96 (0.94–0.97)	0.94 (0.91–0.95)	0.84 (0.79–0.88)	0.265 (0.242–0.289)
Refitting + rcs	0.82 (0.79–0.85)	0.96 (0.94–0.97)	0.94 (0.92–0.96)	0.85 (0.81–0.89)	0.261 (0.237–0.284)
Extension	0.81 (0.78–0.84)	0.96 (0.94–0.97)	0.94 (0.92–0.96)	0.84 (0.80–0.88)	0.262 (0.238–0.287)
Penalized extension	0.81 (0.78–0.84)	0.96 (0.94–0.97)	0.94 (0.92–0.96)	0.84 (0.80–0.88)	0.263 (0.239–0.287)

*PDI* polytomous discrimination index, *FPUL* failed pregnancy of unknown location, *IUP* intra-uterine pregnancy, *EP* ectopic pregnancy, *SGH* St. George’s Hospital, *QCCH* Queen Charlotte’s and Chelsea Hospital, *rcs* restricted cubic splines

### Theoretical results

Due to the non-corresponding linear predictors, the number of coefficients to be estimated in logistic recalibration increases quickly with the number of outcome categories. Logistic recalibration requires the estimation of *k* − 1 intercepts and of (*k* − 1)^2^ coefficients, hence *k* × (*k* − 1) parameters in total. Straightforward refitting of the model requires *k* − 1 intercepts and of *q* × (*k* − 1) coefficients, hence (*q* + 1) × (*k* − 1) parameters in total. This implies that logistic recalibration is obsolete if *q* < *k* because then refitting does not require more parameters.

### Discrimination

Discrimination improved only slightly with more elaborate updating. An interesting finding is that recalibration can affect discrimination, which is not possible for dichotomous risk models. For intercept recalibration, this effect was so small that it was not visible when rounding *c*-statistics to two decimals (Table 4). Logistic recalibration clearly improved discrimination (Table 4). For temporal updating, the PDI increased to 0.88 and the *c*-statistics to 0.93 for FPUL vs EP and 0.91 for IUP vs EP (Table 4). For geographical updating, the PDI remained at 0.80, but *c*-statistics increased to 0.96 (FPUL vs IUP) and 0.93 (FPUL vs EP) (Table 4). Refitting, refitting with inclusion of functional form, and extension led to further small improvements in discrimination. Model extension increased the PDI by +0.01 (geographical updating) or +0.02 (temporal updating) and pairwise *c*-statistics by at most +0.04.

### Calibration

Intercept recalibration improved calibration substantially by correcting calibration-in-the-large (Fig. 2). Due to overfitting of M4, logistic recalibration further improved calibration: the calibration slopes improved to 0.98 (FPUL vs IUP) and 0.97 (EP vs IUP) at temporal updating and to 1 and 0.98 at geographical updating (Fig. 2). Calibration remained good for more elaborate updating methods, although refitting and extension led to slightly lower calibration slopes. This was corrected when penalized versions of these methods were used.

### Overall performance (Brier) and closed testing procedure

The Brier score of the original model was 0.172 at temporal updating and 0.286 at geographical updating (Table 4). This improved gradually when intercept recalibration (0.165 and 0.278), logistic recalibration (0.158 and 0.267), or refitting (0.157 and 0.266) was used. Revision with inclusion of functional form improved Brier scores to 0.153 and 0.263.

Model extension resulted in Brier scores of 0.150 and 0.262. The likelihood ratio tests for the log of progesterone indicated its predictive value at temporal updating (OR FPUL vs IUP = 0.19 (95% CI, 0.12 to 0.30), OR EP vs IUP = 0.30 (0.19 to 0.46) and geographical updating (OR FPUL vs IUP = 0.60 (0.44 to 0.81), OR EP vs IUP = 0.67 (0.50 to 0.91)).

**Fig. 3:**
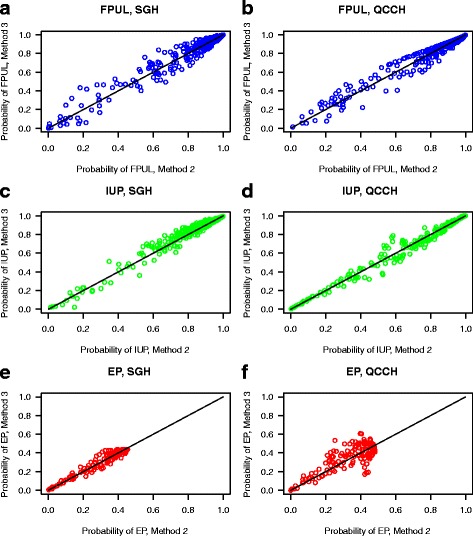
Reclassification plots for logistic recalibration (method 2) vs refitting (method 3) on the temporal (**a**, **c**, **e**) and geographical (**b**, **d**, **f**) updating data: the predicted probability of FPUL when the reference standard is FPUL (**a**, **b**), the predicted probability of IUP when the reference standard is IUP (**c**, **d**), the predicted probability of EP when the reference standard is EP (**e**, **f**)

The closed testing procedure favored refitting over the original model or recalibration methods in both datasets (Table 5), despite minor performance improvements of refitting over logistic recalibration (Table 4, Fig. 2). This suggests that refitting resulted in a better model log-likelihood compared to logistic recalibration and hence that refitting would result in improved predicted risks per patient. This is supported by reclassification graphs [28] shown in Fig. 3: predicted probabilities are on average higher for the true outcome after refitting than after logistic recalibration. For example, in panel C, the predicted risk of IUP for true IUP cases from SGH is often higher after refitting (*y*-axis) than after logistic recalibration (*x*-axis).

**Table 5:** Results of the closed testing procedure

Step	df	Temporal updating (SGH)	Geographical updating (QCCH)
1. Original model vs refitting	8	Δ*ℓ* = 241.6, *p* < 0.0001	Δ*ℓ* = 212.1, *p* < 0.0001
2. Intercept recalibration vs refitting	6	Δ*ℓ* = 169.1, *p* < 0.0001	Δ*ℓ* = 172.7, *p* < 0.0001
3. Logistic recalibration vs refitting	2	Δ*ℓ* = 20.2, *p* < 0.0001	Δ*ℓ* = 22.8, *p* < 0.0001

*df* degrees of freedom, *SGH* St. George’s Hospital, *QCCH* Queen Charlotte’s and Chelsea Hospital, Δ*ℓ* difference in −2 log-likelihood

The updated model coefficients for each method and each dataset are provided in the Appendix.

## Discussion

In this paper, we propose methods to update risk models based on multinomial logistic regression. As a case study, the M4 model to predict the outcome of pregnancies of unknown location [12] was updated temporally (using more recent data from the same setting) and geographically (using data from a different hospital). Seven updating methods were considered: two recalibration methods (intercept recalibration, logistic recalibration), three revision methods (refitting of individual coefficients, penalized refitting of individual coefficients, and refitting with reassessment of functional form of the most important predictor), and two extension methods (straightforward and penalized extension). A closed testing procedure was introduced to select between no updating, intercept recalibration, logistic recalibration, and refitting.

### Conclusions for the case study on the M4 model

The original M4 model was poorly calibrated in both updating settings, but discrimination was very good. Steady but mild improvements in discrimination were observed when increasingly elaborate methods were used. The closed testing procedure suggested refitting in both updating settings. This was likely due to (1) slightly better discrimination, (2) the fact that revision methods should improve the average accuracy of risk predictions per individual, and (3) large validation sample size. Reassessment of functional form appeared to further improve discrimination, whereas penalized refitting had beneficial impact on calibration. Extending the model with progesterone further improved model discrimination.

### Differences between updating of dichotomous vs multinomial risk models

Some dissimilarities can be seen between dichotomous and nominal updating methods. For dichotomous outcomes, recalibration does not change the *c*-statistic, since this is a rank order statistic not affected by linear transformation. A multinomial logistic model contains multiple linear predictors, one for each outcome category vs the reference category. Because of different adaptations to the linear predictors, recalibration methods can change the PDI (nominal *c*-statistic) and the pairwise *c*-statistics.

For multinomial risk models with *k* outcome categories, we have *k* − 1 calibration intercepts and (*k* − 1)^2^ calibration slopes. Hence, logistic recalibration is more complicated for multinomial logistic risk models and in fact even becomes obsolete when *k* is higher than the number of variables *q* in the original risk model (excluding intercepts, but including nonlinear and interaction terms). In these situations, logistic recalibration requires at least as many parameters as straightforward refitting.

In addition, a multinomial calibration plot is more complex than a dichotomous one [13]. For the former, we have one curve for each outcome category while for the latter, a single curve is sufficient. The same predicted risk for one category can be associated with different observed proportions depending on the predicted risks for the other categories [13]. Therefore, irrespective of whether a logistic or flexible calibration analysis is used, smoothing is needed in the calibration plots to visualize the overall relationship between predicted risks and observed proportions.

Finally, the number of parameters to be estimated increases with the number of outcome categories. For example, if the original model has *q* variables, straightforward refitting requires (*q* + 1) × (*k* − 1) parameters. Hence, the more categories, the more cumbersome model revision becomes.

### Choice of updating method

Calibration can often be strongly improved with simple intercept and/or slope adjustments [1, 7, 8]. When updating a prediction model that was originally based on a small sample, as in our case study, intercept recalibration will typically be insufficient as it is likely that the original model is overfitted. Recalibration corrects problems with the calibration intercepts and slopes. This was recently described as “weak” calibration [26]. In contrast, “strong” calibration is defined as the correspondence between predicted probabilities and observed proportions per covariate pattern. This is a utopic goal in empirical studies [26], but if we would like to approach strong calibration, revision methods should be preferred. These methods aim to correct bias in individual model coefficients and hence should on average lead to more accurate predictions per covariate pattern. In our case study, the closed testing procedure indicated that refitting was required although there were minor differences in discrimination and calibration performance measures.

In research on updating methods for risk models, the functional form or optimal transformation of continuous predictors has thus far received limited attention. However, transformations used in the original model may not hold for every setting in which the model may be used. Settings will for example vary with respect to the homogeneity of the patient population, or the transformation used in the original model may be the result of overfitting. Our case study also showed that the functional form of the effect of hCG ratio could be improved from the original model.

In theory, one would always prefer revision methods in order to make optimal adjustments to the model. Computing power will usually not be an issue, but rather the available sample size is a key determinant for the choice of updating method. If sample size is limited, recalibration may already give very good value for money whereas revision may require too much from the available data. However, when sample size is large, model revision will help to further improve discrimination and/or accuracy of predicted risks [26, 29]. Reliably reassessing functional form may require even more data (e.g., updating a linearly modeled covariate with restricted cubic splines). For multinomial models, the number of outcome categories *k* is important as well. If *k* is larger than the number of model variables *q* in the original model, logistic recalibration is obsolete.

Existing evidence for dichotomous risk models recommends at least 100–200 cases in the smallest outcome category for reliable model validation [26, 30, 31]. Further, based on common guidelines for developing dichotomous models, at least 10 cases per coefficient in the smallest outcome category would be recommended to use model revision [1, 32]. The total number of coefficients equals *q* × (*k* − 1) for multinomial updating. For straightforward refitting, 20 cases per coefficient is preferable; otherwise, penalized refitting can be recommended [1]. If sample size is smaller, logistic recalibration is a defendable alternative. However, such guidelines for multinomial risk models require additional research. The closed testing procedure indirectly takes sample size in account by the fact that larger samples yield higher statistical power: for larger samples, the procedure will more easily suggest revision.

### Further research

The influence of sample size (e.g., events per variable (EPV)) on the development, validation, and updating of multinomial logistic models for risk prediction as well as its influence on calibration slopes should be investigated. Second, different penalization techniques for multinomial risk prediction models can be considered, including variants of the Lasso [1, 19, 33–36]. Third, research concerning altering functional form of one or more predictors in case of updating might be conducted. Fourth, it might be of interest to use and consider benchmark values to distinguish between case-mix effects and wrong coefficients when explaining poor validation results of multinomial prediction models [37]. Fifth, updating methods should be evaluated within the context of dynamic/continuous updating, a topic that becomes increasingly relevant [38]. Finally, updating techniques are needed for prediction models for ordinal outcomes.

## Conclusions

Updating methods for dichotomous risk models were successfully adapted to multinomial risk models. Simple recalibration methods may work well even if the original prediction model was based on a relatively small sample. Since the number of parameters to be estimated increases with the number of outcome categories, we recommend full model revision only when the sample size is large. To decide on the appropriate updating complexity, the closed testing procedure is helpful because it will tend to favor recalibration in smaller samples and refitting in larger samples. If the available sample size is large, revision including reassessment of functional form may be considered to better tailor predictions to individual patients or covariate patterns.

## Appendix

1. Exclusion criteria for the case study

For the study on pregnancies of unknown location, exclusion criteria were being lost to follow-up, a missing hCG level at presentation (hCG0) or a level ≤25 IU/L (which is considered as a negative pregnancy test), an unavailable 48-h hCG (hCG48) level, and an hCG48 level that is not taken within 1 to 3 days after the hCG0 level. Some patients do not have an hCG48 level due to an IUP or EP being detected using ultrasonography prior to this blood sample, a very low hCG0 level, failing to return in time, or being lost to follow-up. Of the 2708 patients, 2295 (85%) are available for analysis after applying the exclusion criteria. At SGH, 93 of 1610 women had an hCG0 level ≤25 IU/L, 12 had no hCG48 level, and 83 were lost to follow-up. At QCCH, 1 of 1098 women had no hCG0 level, 17 had an hCG0 level ≤25 IU/L, 125 had no hCG48 level, 22 had hCG48 measured more than 3 days after hCG0, and 60 were lost to follow-up. Therefore, data from 1422 (88%) patients were available at SGH and data from 873 (80%) women at QCCH. At SGH, there were 50% FPUL, 41% IUP, and 9% EP. The QCCH data contained 58% FPUL, 28% IUP, and 14% EP.

2 Logistic recalibration: worked out formula for the case study

Logistic recalibration (method 2) has the following formula for the case study:

$$ \left\{\begin{array}{c}\hfill {\mathrm{LP}}_{2,\mathrm{FvsI}}={\alpha}_1+{\beta}_1\times {\mathrm{LP}}_{\mathrm{FvsI}}+{\gamma}_1\times {\mathrm{LP}}_{\mathrm{EvsI}}\hfill \\ {}\hfill {\mathrm{LP}}_{2,\mathrm{EvsI}}={\alpha}_2+{\gamma}_2\times {\mathrm{LP}}_{\mathrm{FvsI}}+{\beta}_2\times {\mathrm{LP}}_{\mathrm{EvsI}}\hfill \end{array}\right. $$

The linear predictors LP_FvsI_ and LP_EvsI_ for the case study are

$$ \left\{\begin{array}{c}\hfill {\mathrm{LP}}_{\mathrm{FvsI}}= log\left(\frac{P_{\mathrm{FPUL}}}{P_{\mathrm{IUP}}}\right)=5.88-1.18\times log\left(\mathrm{hCGm}\right)-5.56\times \mathrm{hCGrc}+2.05\times {\mathrm{hCGrc}}^2\hfill \\ {}\hfill {\mathrm{LP}}_{\mathrm{EvsI}}= log\left(\frac{P_{\mathrm{EP}}}{P_{\mathrm{IUP}}}\right)=0.39-0.06\times log\left(\mathrm{hCGm}\right)-0.26\times \mathrm{hCGrc}-3.93\times {\mathrm{hCGrc}}^2\hfill \end{array}\right. $$

If we fill in these linear predictors into the logistic recalibration formula, we obtain the following updated linear predictors:

$$ \left\{\begin{array}{cc}\hfill {\mathrm{LP}}_{2,\mathrm{FvsI}}=\hfill & \hfill \left({a}_1+{\beta}_1\times 5.88+{\gamma}_1\times 0.36\right)\hfill \\ {}\hfill \hfill & \hfill +\left({\beta}_1\times -1.18+{\gamma}_1\times -0.06\right)\times log\left(\mathrm{hCGm}\right)\hfill \\ {}\hfill \hfill & \hfill +\left({\beta}_1\times -5.56+{\gamma}_1\times -0.26\right)\times \mathrm{hCGrc}\hfill \\ {}\hfill \hfill & \hfill +\left({\beta}_1\times -2.05+{\gamma}_1\times -3.93\right)\times {\mathrm{hCGrc}}^2\hfill \\ {}\hfill {\mathrm{LP}}_{2,\mathrm{FvsI}}=\hfill & \hfill \left({a}_2+{\gamma}_2\times 5.88+{\beta}_2\times 0.36\right)\hfill \\ {}\hfill \hfill & \hfill +\left({\gamma}_2\times -1.18+{\beta}_2\times -0.06\right)\times log\left(\mathrm{hCGm}\right)\hfill \\ {}\hfill \hfill & \hfill +\left({\gamma}_2\times -5.56+{\beta}_2\times -0.26\right)\times \mathrm{hCGrc}\hfill \\ {}\hfill \hfill & \hfill +\left({\gamma}_2\times -2.05+{\beta}_2\times -3.93\right)\times {\mathrm{hCGrc}}^2\hfill \end{array}\right. $$

3 Updated model coefficients
  **Table:**
  	Parameter of M4				
  Updating method	Intercept	Log(hCGm)	hCGrc	hCGrc^2^	Log(prog)
  Temporal updating (SGH), FP vs IUP					
  No updating	5.88	−1.18	−5.56	2.05	na
  Intercept recalibration	7.07	−1.18	−5.56	2.05	na
  Logistic recalibration	9.12	−1.34	−6.32	1.70	na
  Refitting	6.19	−0.86	−7.37	2.19	na
  Penalized refitting	7.87	−1.14	−6.42	1.74	na
  Refitting + rcs	24.08	−0.87	Replaced by rcs		na
  Extension	10.90	−0.77	−5.49	1.65	−1.67
  Penalized extension	9.84	−0.84	−5.99	1.80	−1.20
  Temporal updating (SGH), EP vs IUP					
  No updating	0.39	−0.06	−0.26	−3.93	na
  Intercept recalibration	0.99	−0.06	−0.26	−3.93	na
  Logistic recalibration	5.29	−0.70	−3.31	−0.36	na
  Refitting	4.01	−0.50	−4.04	0.33	na
  Penalized refitting	4.86	−0.64	−3.33	−0.33	na
  Refitting + rcs	14.44	−0.48	Replaced by rcs		na
  Extension	7.83	−0.47	−2.66	−0.14	−1.21
  Penalized extension	6.83	−0.50	−3.05	−0.05	−0.84
  Geographical updating (QCCH), FP vs IUP					
  No updating	5.88	−1.18	−5.56	2.05	na
  Intercept recalibration	6.15	−1.18	−5.56	2.05	na
  Logistic recalibration	5.08	−0.82	−3.86	0.61	na
  Refitting	4.74	−0.75	−4.04	0.06	na
  Penalized refitting	4.86	−0.78	−3.90	0.50	na
  Refitting + rcs	9.01	−0.73	Replaced by rcs		na
  Extension	5.67	−0.66	−3.38	−0.06	−0.52
  Penalized extension	5.45	−0.73	−3.65	0.38	−0.32
  Geographical updating (QCCH), EP vs IUP					
  No updating	0.39	−0.06	−0.26	−3.93	na
  Intercept recalibration	1.25	−0.06	−0.26	−3.93	na
  Logistic recalibration	1.70	−0.20	−0.91	−1.33	na
  Refitting	3.81	−0.51	−0.42	−1.59	na
  Penalized refitting	3.22	−0.43	−0.67	−1.39	na
  Refitting + rcs	0.79	−0.51	Replaced by rcs		na
  Extension	4.61	−0.45	0.07	−1.75	−0.40
  Penalized extension	3.89	−0.43	−0.41	−1.45	−0.21
  *hCGm* the average of hCG at 48 hours and hCG at presentation, *hCGrc* the centered ratio of hCG at 48 hours and hCG at presentation, *prog* progesterone, *FPUL* failed pregnancy of unknown location, *IUP* intra-uterine pregnancy, *EP* ectopic pregnancy, *SGH* St. George’s Hospital, *QCCH* Queen Charlotte’s and Chelsea Hospital, *rcs* restricted cubic splines
